# *In Vitro* Microtumors Provide a Physiologically Predictive Tool for Breast Cancer Therapeutic Screening

**DOI:** 10.1371/journal.pone.0123312

**Published:** 2015-04-09

**Authors:** Gabriel Benton, Gerald DeGray, Hynda K. Kleinman, Jay George, Irina Arnaoutova

**Affiliations:** Trevigen Inc., Gaithersburg, MD, United States of America; University of Bergen, NORWAY

## Abstract

Many anti-cancer drugs fail in human trials despite showing efficacy in preclinical models. It is clear that the *in vitro* assays involving 2D monoculture do not reflect the complex extracellular matrix, chemical, and cellular microenvironment of the tumor tissue, and this may explain the failure of 2D models to predict clinical efficacy. We first optimized an *in vitro* microtumor model using a tumor-aligned ECM, a tumor-aligned medium, MCF-7 and MDA-MB-231 breast cancer spheroids, human umbilical vein endothelial cells, and human stromal cells to recapitulate the tissue architecture, chemical environment, and cellular organization of a growing and invading tumor. We assayed the microtumor for cell proliferation and invasion in a tumor-aligned extracellular matrix, exhibiting collagen deposition, acidity, glucose deprivation, and hypoxia. We found maximal proliferation and invasion when the multicellular spheroids were cultured in a tumor-aligned medium, having low pH and low glucose, with 10% fetal bovine serum under hypoxic conditions. In a 7-day assay, varying doses of fluorouracil or paclitaxel had differential effects on proliferation for MCF-7 and MDA-MB-231 tumor spheroids in microtumor compared to 2D and 3D monoculture. The microtumors exhibited a tumor morphology and drug response similar to published xenograft data, thus demonstrating a more physiologically predictive *in vitro* model.

## Introduction

It was recently estimated that the average cost to bring a drug to market is now five billion dollars [1]. A major contributing reason for this excessive cost is from the early preclinical work which shows initial promise and then collapses in clinical trials. To properly identify the most efficacious drug candidates and reduce the cost of development, drug screening strategies are needed that more accurately predict patient response, and this can be accomplished by developing assay systems that exhibit extracellular matrix (ECM), biochemical, and cellular features of tumor tissue.

There has been much work done to understand key features of the tumor microenvironment that are important for determining tumor architecture and activity. First of all, the composition and mechanical properties of the ECM are important for morphology, gene expression, proliferation, invasion, and drug response for cancer cells. When cultured in ECM hydrogels, breast cancer cells assemble into physiological three dimensional structures that are representative of their malignant potential and correlate with their gene expression profiles. Low metastatic cells grow in tight clumps; whereas, malignant cells will form processes that invade into the surrounding ECM [2, 3]. When collagen-1 deposition is increased within hydrogels composed of the basement membrane protein laminin-1, DNA methyltransferase-1 expression is upregulated, increasing epigenetic DNA methylation and eliciting a more invasive breast cancer phenotype [4]. Also, increasing collagen-1 concentrations enhances matrix stiffness which modulates cell invasion and paclitaxel drug response for breast cancer spheroids [5]. Secondly, the tumor microenvironment can be further characterized biochemically as a low oxygen, low pH, and low glucose environment [6, 7]. It has been shown that hypoxia increases the invasiveness of prostate cancer and squamous cell cancer cell lines in 3D culture [8, 9], and by incorporating the low oxygen, low pH, and low glucose environment into 3D cultures, the expression of inducers of breast cancer cell proliferation and metastasis, such as interleukin-6, is promoted [10]. Finally, within the tumor microenvironment, other cell populations provide essential elements, in the form of secreted ECM proteins, cell-to-cell contacts, and soluble factors, that direct tumor tissue activities. Cancer cell proliferation may be promoted by endothelial cells [11], and the presence of adipose-derived stem cells increases proliferation, migration, invasion, and chemoresistance for cancer cells [12–14]. Thus, the ECM, biochemical, and cell populations contribute to tumor tissue activities, and their proper integration into *in vitro* assays provides for more physiologically predictive responses.

The development of these assays has entailed the gradual evolution of cell culture techniques. The current *in vitro* testing that is carried out on either plastic dishes or on agar, also known as the colony forming assay, using tumor cell lines in monoculture has not change significantly in decades. These systems lack the physiologic ECM, tumor architecture, biochemical environment, and other cell types found within tissues. Initial attempts at recreating tumor physiology using an exogenous ECM have held promise [15]. Cancer cells dispersed within a physiological ECM hydrogel to form 3D cultures exhibit key features of early tumor structure and gene expression [16]; however, these models lack the size and organization of more mature tumors, which are prevalent at the time of diagnosis [17]. Cancer cells can be directed to adopt the appropriate *in vivo* tumor morphology as multicellular tumor spheroids (MCTS), that exhibited physiological gradients for pH, oxygen, waste, and nutrients [18]. These gradients establish heterogeneous cell populations within the MCTS that exhibit different responses to radiation and chemotherapy [19–21], and malignant cancer cells can invade out of the MCTS into an ECM hydrogel, recreating early metastatic events [22]. Since most solid tumors are derived from and grow within tissues, it is no surprise that the introduction of other cell types from these emanating tissues influence proliferation and drug resistance in these MCTS [13, 23, 24]. It can thus be predicted that modeling the tumor microenvironment by recreating the ECM composition, biochemical properties, cell populations, and tissue architecture will provide a model system that has more authentic responses to anti-cancer treatments.

To this end, we have optimized a tumor-aligned *in vitro* microtumor model using breast cancer cell spheroids, endothelial cell tubules, and human stromal cells cultured in an extracellular matrix composed of basement membrane proteins and collagen-1 which is acidic, hypoxic, and glucose-deprived. To validate this model, we evaluate the morphology, proliferation, invasion, and anti-cancer drug response of the microtumors. Based on parallel studies in both 2D and 3D monoculture under non-tumor-aligned conditions, we conclude that this new *in vitro* microtumor method is an improved system for anti-cancer drug screening.

## Methods

### Materials

Human breast cancer cell lines MCF-7 and MDA-MB-231 were obtained from ATCC (Manassas, VA). Cancer cell lines were transfected with pVision-RFP-C Vector from BioVisions (Milpitas, CA) using Transfast from Promega (Madison, WI). Human umbilical vein endothelial cells (HUVECs) and EGM-2 medium were obtained from Lonza (Walkersville, MD). Adipose-derived, human mesenchymal stem cells (hMSC) and hMSC medium were obtained from ATCC (Manassas, VA). The basement membrane extract (BME), tumor-aligned basement membrane extract Type 3 (BME-3), 10X Spheroid Formation ECM, Tumor-aligned RPMI (TARPMI), collagen-1, and calcein am were obtained from Trevigen, Inc. (Gaithersburg, MD). RPMI 1640 was obtained from Invitrogen (Carlsbad, CA). Tissue culture flasks, 96 well ultra-low adhesion plates, and 96 well tissue culture-treated plates were obtained from Corning, Inc. (Corning, NY). Fetal bovine serum (FBS) was obtained from HyClone (Logan, UT). Fluorouracil and paclitaxel were both identified as current breast cancer treatments from the NCI Developmental Therapeutics Program [DTP] Approved Oncology Drugs Set, and these and ethidium bromide were obtained from Sigma (St. Louis, MO). The cytoplasmic membrane dyes, Neuro-DiO and DiB, were obtained from Biotium (Hayward, CA).

### Cell Culture Maintenance

MDA-MB-231 and MCF-7 cells were cultured in RPMI, containing 10% FBS, and passaged at 1:5 when they reach 80% confluence. HUVECs were cultured in EGM-2 defined medium and passaged 1:4 when they reach 80% confluence. hMSCs were cultured in MEM, containing 10% FBS, and passaged 1:2 when they reach 90% confluence. Cells were cultured at 37°C, 5% CO_2._

### 2D Monoculture

Breast cancer cells were seeded at 2,000 cells per well in a 96 well plate, and allowed to adhere for 24 hours prior to treatment or analysis. Samples were evaluated in quadruplicate.

### 3D Monoculture

Each well of a 96 well plate was coated with 50 μl of BME at 4°C. The plate was centrifuged 300 *x g* at 4°C for 10 minutes to eliminate bubbles. The plate was then placed in a cell culture incubator at 37°C, 5% CO_2_ for 30 minutes to promote hydrogel polymerization. Then 2,000 harvested breast cancer cells were seeded in each well in RPMI, 10% FBS, 2% BME cell culture medium. The plate was incubated at 37°C, 5% CO_2_ for 96 hours prior to treatment or analysis. Samples were evaluated in quadruplicate.

### Spheroid Cultures

**Fig 1:**
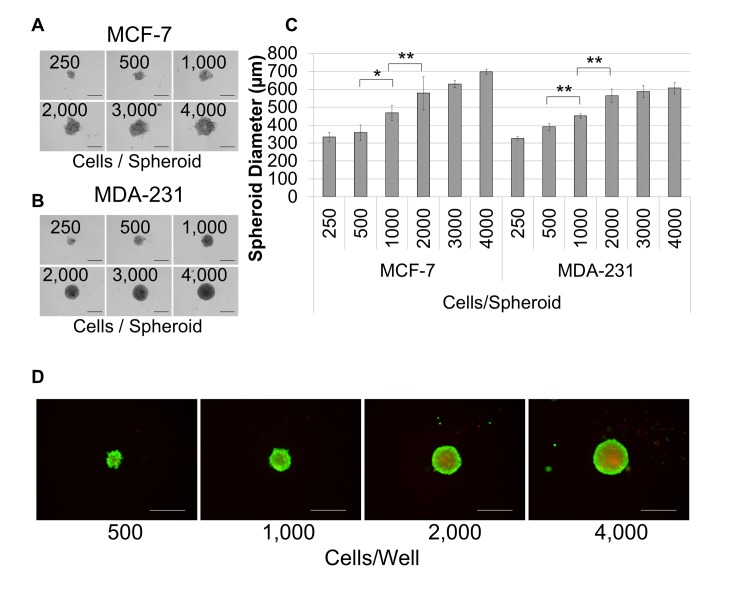
The optimal breast cancer cell seeding density for spheroid formation is 2,000 cells/well. Breast cancer cells were seeded at the concentrations (cells/well) shown in 96 well, ultra-low adhesion plates and incubated for 72 hours in 1X spheroid formation ECM for MCF-7 (A) and MDA-MB-231 (B) cells. C. The diameter of each spheroid was measured for quadruplicate samples and groups were compared for statistical significance using the student’s *t*-test for both cell lines. D. Non-fluorescent calcein am was converted to fluorescent calcein (green), indicating living cells, and ethidium bromide (red) was internalized by dead cells. The presence of live cells in the outer layers of the spheroid and dead cells in the spheroid core is indicative of physiological diffusion gradients. Scale bar = 500 μm. *P < 0.05, **P < 0.01.

**Fig 2:**
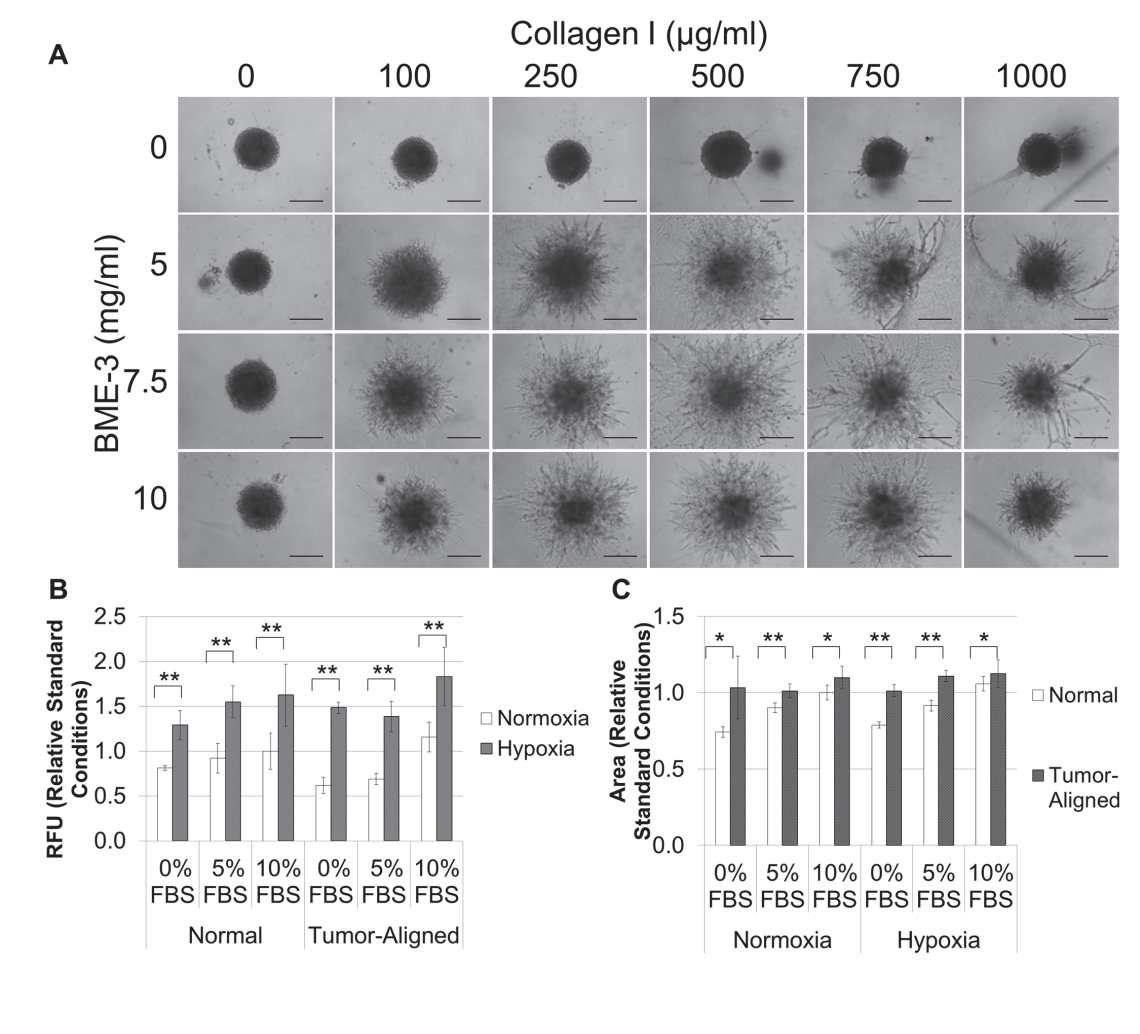
The optimal Invasion Matrix is 10 mg/ml BME, 250 μg/ml collagen-1, and MDA-MB-231 spheroids exhibit maximum proliferation and invasion when cultured in a tumor-aligned medium with 10% FBS under hypoxia. A. MDA-MB-231 spheroids formed from 2,000 cells/well were embedded in hydrogels composed of different mixtures of BME (0, 5, 7.5, and 10 mg/ml) and collagen (0, 100, 250, and 750 μg/ml collagen-1). After polymerization, 100 ul of RPMI, 10% FBS was added to each well to elicit an invasive response and determine optimal matrix composition for invasion of cells out of the spheroid and into the surrounding matrix. B and C. Spheroids were formed as described above. After 72 hours, 50 μl of Invasion matrix or tumor-aligned invasion matrix were added to each well, and the plates were incubated at for 1 hour to polymerize hydrogel. Then, an additional 100 μl of medium (normal or tumor-aligned, as indicated above) containing the amount of FBS indicated was added to each well, and plates were incubated in either a normal cell culture incubator or a hypoxia chamber, as indicated above. Plates were read in a 96 well plate reader at excitation 540 nm/ 587 nm emission to compare proliferation of breast cancer cells (B), and spheroids were photographed using the TRITC filter to compare invasion of breast cancer cells (C). Each condition was assessed in quadruplicate after 96 hours. Photographs were analyzed using ImageJ to determine structure size which reflected cell invasion. Scale bar = 500 μm. *P < 0.05, **P < 0.01.

**Fig 3:**
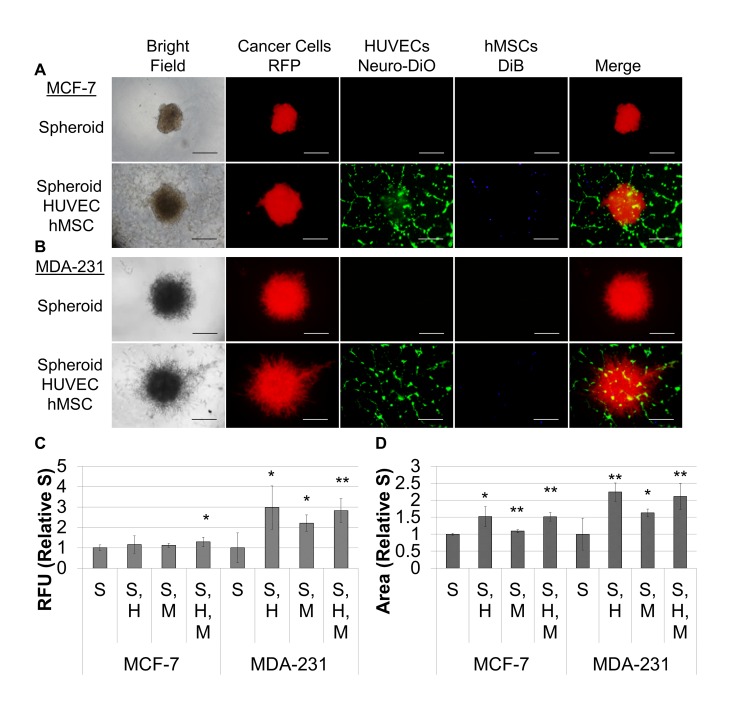
hMSCs and endothelial tubules promote invasion and differentially effect proliferation of MDA-MB-231 and MCF-7 spheroids. A. Spheroids formed as described above under hypoxia. Then 96 well, flat bottom plates were coated with 50 μl of tubule formation matrix and incubated for one hour to polymerize the hydrogel. For wells with HUVECs, 12,500 cells were added to each well, and for remaining samples, EGM-2 was added. HUVECs were allowed to assemble into tubules for two hours. One spheroid was transferred to each of the wells in the plate. MCTS were allowed to settle for 1 hour. Then, 100 μl of medium was aspirated from each well. For wells with hMSCs, 10,000 cells were suspended in each ml of Invasion Matrix, and 50 μl was added to each well. For the remaining samples, 50 μl of tumor-aligned invasion matrix was added to each well. The plates were then incubated at 37°C, 5% CO_2_ for 1 hour to polymerize the hydrogel, and 100 μl of TARPMI, 10% FBS was added to each well. Cultures were incubated under hypoxia for 96 hours. Images are provided for spheroids alone and for spheroids, HUVECs, and hMSCs for MCF-7 (A) and MDA-231 (B). Cultures were analyzed as described above for proliferation (C) and invasion (D), and samples were evaluated in quadruplicate. S = breast cancer MCTS, H = HUVEC network, and M = hMSCs. Scale bar = 500 μm. *P < 0.05, **P < 0.01.

Breast cancer cells were harvested, diluted as indicated in RPMI, 10% FBS, 1X Spheroid Formation ECM, seeded at 50 μl/well in 96 well ultra-low adhesion plates, and incubated for 72 hour at 37°C, 5% CO_2_ to promote spheroid formation. The resulting spheroids could be photographed and measured (Fig 1A–1C). Alternatively, they could be embedded in ECM to assess environmental conditions. They were embedded in extracellular matrix composed of BME-3 (0, 5, 7.5, or 10 mg/ml) and Collagen I (0, 100, 250, 500, 750, and 1,000 μg/ml) (Fig 2A), or they were embedded in 10 mg/ml BME, 250 ug/ml Collagen I (Invasion Matrix) and evaluated for the effects of oxygen (normoxia = 37°C, 5% CO_2,_ 19% O_2_; hypoxia = 37°C, 5% CO_2_, 5% O_2_), normal (RPMI) or tumor-aligned (TARPMI) medium, and FBS content (0%, 5%, or 10%) on proliferation (Fig 2B) and invasion (Fig 2C). Spheroids were also embedded in Invasion matrix alone or with endothelial networks, stromal cells, or a combination of the two under tumor-aligned conditions (Fig 3A and 3B) to determine the impact on cancer cell proliferation (Fig 3C) and invasion (Fig 3D). Spheroids were embedded for 96 hours unless otherwise indicated. Samples were evaluated in quadruplicate.

### Endothelial Tubule Formation

Each well of a 96 well plate was coated with 50 μl of BME/collagen-1 mix at 4°C. HUVECs were labeled with NeuroDio. HUVECs were then harvested, and 15,000 cells were seeded in each insert in EGM-2 cell culture medium. HUVECs were allowed to assemble into tubules for two hours. hMSCs were labeled with DiB. hMSCs were harvested, and 3,000 hMSCs were added to each well. The plate was incubated at 37°C, 5% CO_2_ for one hour prior to addition of the tumor-aligned spheroid (see below).

### In Vitro Microtumors

Breast cancer cells and hMSCs were harvested, diluted to 40,000 cells/ml and to 10,000 cells/ml, respectively in TARPMI, 10% FBS, 1X Spheroid Formation ECM, seeded at 50 μl/well in 96 well ultra-low adhesion plates, and incubated for 72 hour at 37°C, 5% CO_2_ to promote spheroid formation. At this time, spheroids were transferred to a 96 well plate containing HUVEC tubules, and embedded in 50 μl tumor-aligned BME-3/Collagen-1 mix. The embedded cultures were then incubated at 37°C, 5% CO_2_ for one hour to promote hydrogel polymerization. The embedded culture was then overlaid with 100 μl TARPMI containing 10% FBS with or without drug treatment. Each well was photographed every 24 hours for image analysis. Samples were evaluated in quadruplicate.

### Live/Dead Cell Assay

MDA-MB-231 breast cancer spheroids were incubated with 2 μM calcein am and 1 μM ethidium bromide in RPMI, 10% FBS for 15 minutes and visualized using epifluorescence [25]. Images were captured using FITC (calcein) and TRICT (ethidium bromide) and merged.

### Cell Proliferation

**Fig 4:**
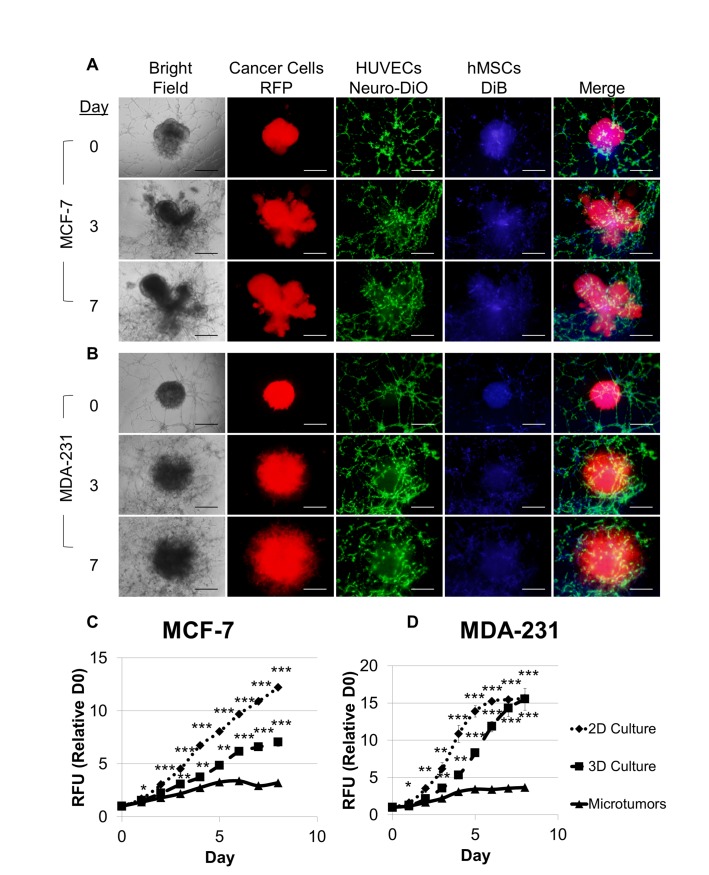
Microtumors of MCTS, hMSC, and endothelial tubules produce a physiological breast cancer niche possessing tumor morphology, tumor invasion, and endothelial recruitment and exhibiting differential cell proliferation compared to 2D and 3D monocultures. Microtumors were modified from Fig 3 such that hMSCs at 1,000 cells/well were added first to the HUVECs that had been incubated for 2 hours. After one hour of HUVEC/hMSC coculture, one MCF-7 spheroid (A) or MDA-MB-231 spheroid (B) containing 500 cells/well hMSCs was added to each well, embedded in TA Invasion Matrix, and overlaid with TARPMI, 10% FBS. A direct comparison of cell proliferation was made between microtumors, 2D monoculture, and 3D monoculture based on fluorescence intensity of the RPF-expressing MCF-7 (C) and MDA-MB-231 (D) cells over eight days in culture. Values were normalized to fluorescence intensity of the onset of assay for each cell line and culture condition, and samples were evaluated in quadruplicate. Scale bar = 500 μm. *P < 0.05, **P < 0.01, ***P < 0.001.

Breast cancer cells expressed red fluorescent protein (RFP). The fluorescence intensity for each well was measured at 540 nm/ 587 nm emission using a Tecan Infinite M200 to determine relative fluorescent units (RFU) for each sample. Controls were prepared in each plate using the same medium, ECM proteins, and/or non-cancer cells (HUVECs and/or hMSCs), to determine background fluorescence, and the background RFU were subtract from each sample RFU. For comparison of spheroid growth in response to normoxic or hypoxic conditions with different concentrations of FBS in normal or tumor-aligned culture medium (Fig 2B), the RFUs for each sample were normalized to the average RFUs for spheroids grown under standard conditions (normoxia, normal medium with 10% FBS). For comparison of the impact of the presence of additional cell types on proliferation of breast cancer cells (Fig 3C), the RFUs for each sample were normalized to the average RFUs for breast cancer spheroids alone (no other cell types present). For comparison of proliferation for 2D culture, 3D culture, and microtumors over an eight day period (Fig 4C), the RFUs for each sample were normalized to the average RFUs for each culture condition at the onset of the culture (day 0). Samples were evaluated in quadruplicate.

### Cell Invasion

Breast cancer cells expressed red fluorescent protein (RFP), and they were visualized via fluorescence microscopy using a TRITC channel filter; whereas, neither HUVECs (NeuroDio) nor hMSCs (DiB) were visible under this filter. The largest area from breast cancer spheroids was photographed and subjected to image analysis using ImageJ. The software was calibrated to the image to convert pixels to unit measure (μm) and to calculate the area (μm^2^) occupied by breast cancer cells. The increase in the area of breast cancer cells alongside the change in morphology from a compact sphere to projecting branches or lobes was indicative of invasion. Samples were evaluated in quadruplicate. A figure describing the procedure for analyzing cell invasion is provided (S1 Fig).

### Cell Labeling

HUVECs and hMSCs were labeled with NeuroDio (green) and DiB (blue) fluorescent dyes, respectively, according to the manufacturer’s instructions. Cells were culture to 80% confluence. The cell culture medium was aspirated and replaced with complete cell culture medium containing a 1:200 dilution of fluorescent dye. The cells were incubated with the diluted dye for 20 minutes at 37°C, 5% CO_2_, and then the cells were washed three times with warm growth medium, incubating cells for 5 minutes in each wash at 37°C, 5% CO_2_. The cells were harvested, resuspended in growth medium, counted, evaluated for viability by trypan blue exclusion, resuspended at 1 x 10^6^ cells/ml, and diluted as needed for assays.

### Image Analysis

Images were captured using an Olympus IX51 with epifluorescence capabilities. Digital photographs were analyzed using ImageJ image analysis software[26]. The software was calibrated to the image to convert pixels to μm. For spheroid invasion analysis, the area of labeled cells from the largest focal plane was used.

## Results

First spheroid formation was optimized for two different breast cancer cell lines, luminal A, MCF-7, cells and claudin-low, MDA-MB-231, cells [27]. The MCTS model provides for tumor size, architecture, and physiology for both MCF-7 (Fig 1A) and MDA-MB-231 (Fig 1B) cell models. Optimal seeding densities of 2,000 cells/well were established for the breast cancer cells that provided a spheroid diameter that was greater than 400 μm (Fig 1C), which is sufficient for the establishment of physiological gradients for pH, oxygen, nutrients, and waste [28]. These gradients were verified by using an assay to differentiate living from dead cells. Here, the conversion of non-fluorescent calcein am to fluorescent calcein which can be visualized in the FITC channel (green), indicates living cells, and the ability of ethidium bromide to penetrate through the plasma and nuclear membranes into the nucleus and intercalate into the DNA, which can be visualized in the TRITC channel (red), indicates dead cells (Fig 1D). The presence of living cells in the out layers and dead cells in the inner layers of the spheroid is indicative of these physiological gradients where the limited diffusion of nutrients in and waste out is detrimental to cell health. Spheroids compose of 2,000 cells/well provided adequate size and exhibited physiological gradient, so this value was used for all future studies.

We next evaluated the optimal invasion matrix. We empirically determined the optimal matrix configuration using BME-3 supplemented with various amounts of collagen-1 as it is known that collagen-1 deposition contributes to matrix stiffening and the malignant phenotype [29]. We found that 10 mg/ml BME-3 and 250 μg/ml of collagen-1 elicited optimal invasion and defined this mixture as our Invasion Matrix (Fig 2A). Then, we evaluated the proliferation and invasion of the MDA-MB-231 cells grown in either normal or tumor-aligned media, which has a low pH and a low glucose concentration, under either normal or hypoxic conditions. Various amounts of fetal bovine serum (FBS) were also included in the assays to determine the optimal spheroid response. Hypoxia clearly increased cell proliferation at all FBS levels tested under both normal and tumor-aligned conditions (Fig 2B), and medium containing 10% FBS was found to be optimal for spheroid cell proliferation. Invasion under both normoxic and hypoxic conditions was similar, and the tumor-aligned medium clearly promoted increased invasion at all levels of FBS (Fig 2C). Again, medium containing 10% FBS was optimal for invasion. These findings showed that maximum proliferation and invasion occurred when the spheroids were cultured in tumor-aligned medium with 10% FBS under hypoxic conditions.

Then, we considered the effect of endothelial cells and hMSCs on proliferation and invasion of the MCF-7 and MDA-MB-231 tumor cells in the Invasion Matrix in tumor-aligned medium under hypoxia. Since the simple addition of endothelial cells to the MCTS was insufficient to promote vascular structure (data not shown), we used fully formed tubules. A fully formed breast cancer cell MCTS was placed on top of preformed endothelial tubules, and these structures were embedded within Invasion Matrix containing hMSCs (Fig 3A and 3B). MCF-7 spheroids exhibited a significant increase in proliferation only in the presence of both hMSCs and endothelial tubules, while the addition of either hMSCs, endothelial, or the combination of the two increased proliferation for MDA-MB-231 spheroids (Fig 3C). Similarly, the incorporation of hMSCs, endothelial tubules, or their combination was sufficient to increase invasion for both MCF-7 and MDA-MB-231 spheroids (Fig 3D).

While there was a functional response for the breast cancer cells when the other cell types were present, the MCTS lacked certain morphological features that are present in tumors; however, we determined that providing the appropriate cell-to-cell interactions promoted the tumor phenotype. The MCF-7 spheroids (Fig 3A) retained the spheroid morphology after embedding in the Invasion Matrix; whereas, the addition of hMSCs during spheroid formation promoted lobular formation and spreading for the MCF-7 (Fig 4A). Similarly, the MDA-MB-231 spheroids exhibited a more directed invasion pattern (Fig 3B); whereas, the addition of hMSCs during spheroid formation promoted a denser, more curved invasion pattern (Fig 4B). Furthermore, dispersion of the hMSCs in the Invasion Matrix was insufficient to stabilize or elicit any response in the tubule networks (Fig 3A and 3B); however, addition of the hMSCs directly to the aligned HUVEC tubules allowed the hMSCs to colocalize to the HUVEC network and promote stabilization and recruitment of the vascular network to the expanding spheroid (Fig 4A and 4B). A detailed diagram explaining the optimized procedure for microtumor assembly is provided in S2 Fig.

Next, the optimized microtumors were compared with 2D and 3D monoculture, and differences in breast cancer cell proliferation corresponded with the differences in cell morphology for 2D culture, 3D culture and microtumors (S3 Fig). After eight days, there were approximately twice as many MCF-7 cells in the 3D culture compared to the microtumor, and there were approximately three times as many MCF-7 cells in the 2D culture compared to the microtumors (Fig 4C). For MDA-MB-231 cells, there were approximately three times as many cells in both the 2D and 3D monocultures compared to the microtumors after eight days; however, proliferation of the 2D culture exhibited a greater rate of increase (Fig 4D). Adding the preformed spheroids containing both breast cancer cells and hMSCs to endothelial tubules composed of both HUVECS and hMSCs provided an improved cell environment, promoting physiological architecture and activity for each microtumor model.

**Fig 5:**
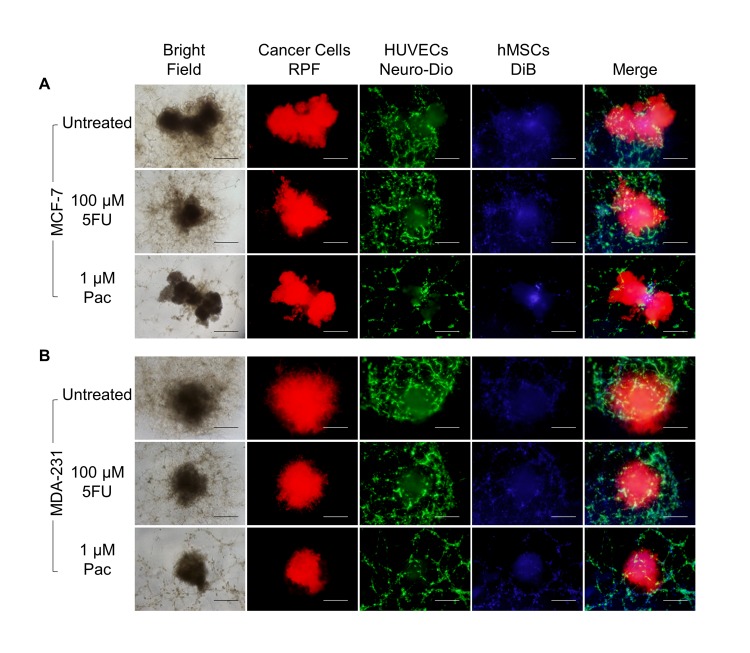
Fluorouracil and paclitaxel differentially affect the morphology of the structural elements of the breast cancer microtumors. Microtumors were established and treated as described above under hypoxia and analyzed on day 7. A. MCF-7 cells exhibit a lobular morphology with fluorouracil or paclitaxel treatment with no apparent change in size. B. MDA-MB-231 cells exhibit an invasive morphology with a decrease in the number and extent of protrusions upon treatment with either fluorouracil or paclitaxel. For both microtumor models, endothelial recruitment is only inhibited in the presence of paclitaxel. Samples were evaluated in quadruplicate. Scale bar = 500 μm. *P < 0.05, **P < 0.01.

Next, we evaluated the effects of two standard chemotherapeutic agents, fluorouracil and paclitaxel, on the morphology of the microtumors over seven days in culture. There was no obvious change in MCF-7 microtumor size or structure with either fluorouracil or paclitaxel at physiological attainable dosages of 100 μM and 1 μM, respectively; however, there was an obvious effect on the integrity of the endothelial tubule networks only for paclitaxel (Fig 5A). Alternatively, the MDA-MB-231 microtumors exhibited an evident decrease in size and spread with either fluorouracil or paclitaxel treatment; and there was an obvious deterioration of the endothelial networks only upon paclitaxel treatment (Fig 5B), similar to MCF-7 microtumors. This phenotypic analysis provides important insight into the impact of treatment on the structural organization and integrity of the individual cell types of the microtumors.

**Fig 6:**
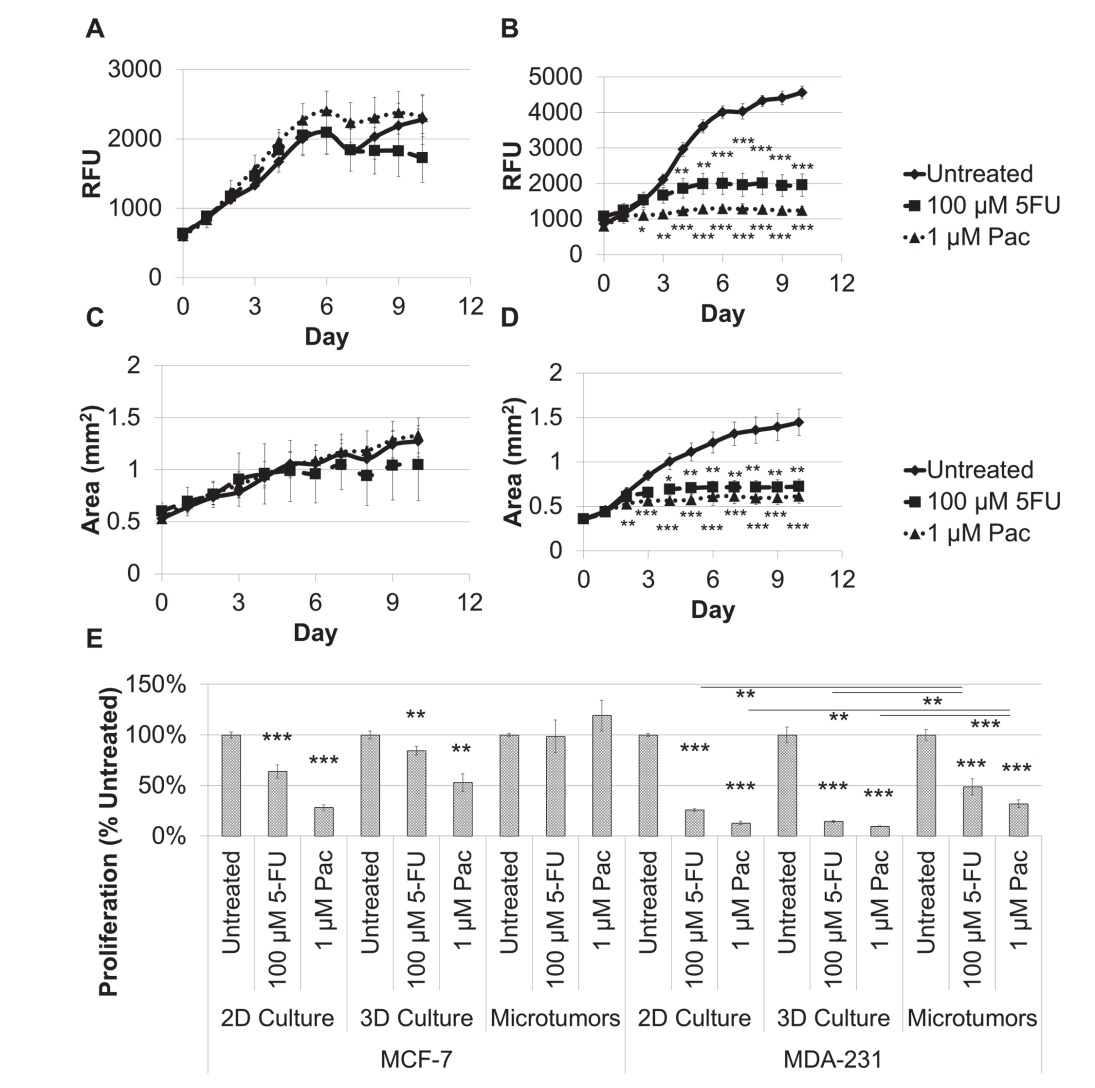
Microtumors exhibit physiological responses to paclitaxel or fluorouracil treatment, and microtumors exhibit a decrease in breast cancer cell proliferation compared to 2D and 3D monocultures for MCF-7 and MDA-MB-231 cells after 7 days of treatment. Microtumor growth and response to 100 μM fluorouracil or 1 μM paclitaxel were determined based on fluorescence intensity of the RPF-expressing MCF-7 (A) and MDA-MB-231 (B) cells over ten days. A similar response was observed for the increase in microtumor area as an indicator of spread or invasion for these treatments for the MCF-7 (C) and MDA-MB-231 (D) microtumors. E. A direct comparison was made between 2D culture, 3D culture, and microtumors on day 7 for MCF-7 and MDA-MB-231 in the presence of fluorouracil or paclitaxel. Fluorouracil and paclitaxel inhibit breast cancer cell proliferation and spread for MDA-MB-231 microtumors, but not for MCF-7 microtumors. In addition, 2D and 3D monocultures were hypersensitive to fluorouracil or paclitaxel treatment compared to microtumor for both cell lines. Samples were evaluated in quadruplicate. *P < 0.05, **P < 0.01, ***P < 0.001.

Similar to the results described above (Fig 5A), there was no significant change in proliferation for the tumor cells in the MCF-7 microtumors for treatment with either fluorouracil or paclitaxel (Fig 6A), while the MDA-MB-231 microtumors exhibit growth inhibition in response to both treatments within the first three days (Fig 6B). In addition, the area for the MCF-7 microtumors was unaffected by treatment (Fig 6C), while there was a substantial increase in area only for the untreated MDA-MB-231 microtumors (Fig 6D). To make a direct comparison of proliferation after treatment between 2D culture or 3D culture and the microtumors when treated with fluorouracil or paclitaxel, the fluorescence intensity for the RFP-expressing cancer cells was normalized to the untreated sample within each culture for each treatment after 7 days (Fig 6E). For MCF-7 models, there was a decrease in cell proliferation compared to untreated cultures for both fluorouracil and paclitaxel for 2D culture (64% and 28%, respectively) and for 3D culture (84% and 53%, respectively) but not for microtumors. For MDA-MB-231 models, there was a decrease in cell proliferation compared to untreated cultures for fluorouracil and for paclitaxel for 2D culture (26% and 13%, respectively), for 3D culture (14% and 10%, respectively), and for microtumors (49% and 32%, respectively). These data demonstrate the more selective resistance to anti-cancer drug treatment for the microtumor model over that in 2D and 3D monoculture.

## Discussion

We have developed *in vitro* microtumors composed of breast cancer cell spheroids, endothelial tubules, and stromal stem cells in an ECM under physiological tumor conditions. Metastatic cancer cells invade out of the MCTS into a surrounding ECM hydrogel, recapitulating early events for local cancer spread [30]. The composition of the ECM is important; the basement membrane proteins induce a tumor phenotype with the formation of cell-cell bonds. The incorporation of collagen-1 elicits an invasive branching phenotype; whereas, culture on collagen-1 alone results in migrating single cells [4]. While the addition of collagen to basement membrane proteins promotes the malignant response, too much collagen can create a barrier that stifles this response [5]. We defined the ECM composition that elicits the most robust invasive response from the MCTS.

We further optimized the biochemical environment for the MCTS based on changes in oxygen, pH, glucose, and FBS concentrations. Hypoxia is a common feature of advanced tumors that leads to tumor survival and progression [31]. Hypoxia promotes angiogenesis, cell survival, proliferation, and invasion [32]. Hypoxia increases the expression of several gene products, including but not limited to HIF-1apha, Sp1 transcription factor, lysyl oxidase (LOX), prolyl hydroxylase-3 (PHD3), heparanase, MMP-2, alpha5 integrin, fibronectin, and QSOX1 [8, 9, 33–39]. The essential role of some of these proteins, including HIF-1alpha, Sp1, LOX, heparanase, alpha5 integrin, fibronectin, and QSOX1, in hypoxia-mediated invasion has been demonstrated. Protein expression may be HIF-1alpha dependent (QSOX1, alpha5 integrin, fibronectin, MMP-2), or it may be independent of HIF-1alpha (LOX). HIF-1alpha also binds to the two hypoxia-response elements of QSOX1 [39]. Interestingly, knockdown of LOX or inhibition with β-amioproprionitrile (an inhibitor of LOX) down regulates HIF-1alpha and decreases invasion [34]. Hypoxia had the greatest effect on breast cancer spheroid growth in our model, probably owing to the presence of the exogenous ECM.

Tumors also grow in an acidic environment due to their high metabolic rate and the release of many degraded factors. The acidity of tumors actively contributes to tumor growth and progression and is associated with a poor patient prognosis [40]. Furthermore, an acidic environment is associated with resistance to both chemotherapy and radiation, suppression of cytotoxic lymphocytes, loss of the tumoricidal activity of natural killer cells, generation of pluripotent stem cells, invasiveness, epithelial-to-mesenchymal transition (EMT), angiogenesis, lymph angiogenesis, and resistance to apoptosis [40]. As a result of the high metabolic requirement of the growing tumor, glucose deprivation is another characteristic of the tumor microenvironment. Colorectal cancer cells with either a KRAS or BRAF mutation exhibited high expression levels of the glucose transporter-1 (*GLUT1*) gene and were able to survive in low glucose environments; whereas, the wild type cells exhibited low levels of *GLUT1* and were not viable in low glucose conditions [41]. The optimal conditions for the physiological breast cancer spheroid growth and invasion model developed here mimic the *in vivo* tumor microenvironment that is hypoxic, acidic, and glucose-deprived.

Tumors are not composed solely of cancer cells, and to recapitulate tumor tissue, hMSCs, endothelial cells, or the combination of the two were integrated into the culture in a manner providing physiological architecture and activity. Stromal cells enhance breast cancer cell proliferation in a cell line-dependent manner [42]. We found that the incorporation of hMSCs alone into the tumor spheroid was sufficient to increase proliferation of MDA-MB-231 cells but did not affect MCF-7 cells; whereas, when the MCTS was cultured in the presence of both endothelial tubules and hMSCs, there was an increase in breast cancer cell proliferation for both cell lines (Fig 3C). When MCF7S1 breast cancer cells were cultured with mammary fibroblasts in 3D culture, the fibroblasts increased expression of the S100A4 metastasis promoting protein, which subsequently increased MMP-2 activity and cytokine secretion within the coculture to promote breast cancer cell invasion [43]. Likewise, we observed an increase in spheroid invasion for both MCF-7 and MDA-MB-231 in the presence of hMSCs, as well as in the presence of endothelial tubules or a combination of the two (Fig 3D). Therefore, the incorporation of hMSC within the MCTS directed the development of tumor architecture with the MCF-7 spheroids assuming a multi-lobular phenotype (Fig 4A) and the MDA-MB-231 spheroids assuming a wavy, invasive phenotype (Fig 4B) within the Invasion Matrix, similar to what has been reported for xenograft models for both cell lines [14, 44]. HUVECs were assembled into tubules on an ECM hydrogel separate from MCTS, as discussed previously [45]. The addition of hMSCs to HUVEC networks promotes survival, migration, and angiogenesis, providing both vascular stability and activity [46], and this activity was reflected in our model for both breast cancer cells. Similar to *in vivo* studies, the microtumors also exhibit much slower rates of proliferation compared to 2D and 3D monocultures for both breast cancer cells (Fig 4C and 4D), indicative of a more physiological metabolism and cell cycle.

Microtumors provide more physiologically predictive responses to chemotherapeutic treatment compared to 2D and 3D cultures. Fluorouracil irreversibly inhibits thymidylate synthase, blocking thymidine production and subsequent DNA replication, and its maximum plasma concentration after administration (Cmax) is between 38 μM and 384 μM [47]. Another commonly used chemotherapeutic, paclitaxel, is a mitotic inhibitor that stabilizes cellular microtubules, preventing metaphase spindle configuration and blocking cell division, and its Cmax is between 0.228 μM and 4.27 μM [48]. By exposing breast cancer microtumors to physiologically attainable dosages of 100 μM fluorouracil or 1 μM paclitaxel and assessing the fluorescence intensity every 24 hours, we can determine microtumor drug response over time similar to *in vivo* tumor growth. MCF-7 and MDA-MB-231 microtumors exhibit the predicted response to physiologically achievable dosages of both fluorouracil and paclitaxel based on xenograft models using the same breast cancer cell lines [49–51]. MCF-7 microtumors were resistant to fluorouracil or paclitaxel treatment (Fig 6A), while MDA-MB-231 microtumors were responsive to both treatments (Fig 6B). The same outcomes were arrived at when using phenotypic analysis to measure the increase in area over time for MCF-7 (Fig 6C) and MDA-MB-231 (Fig 6D) microtumors.

There is much evidence to suggest that different culture methods elicit differential drug responses using the same cell lines [52]. The response to paclitaxel treatment elicited by 2D monocultures is not representative of *in vivo* drug response and is hypersensitive for MCF-7 and MDA-MB-231 breast cancer cell lines [50]. Our observation was similar; the 2D and 3D monocultures exhibit hypersensitivity to physiological attainable dosages of fluorouracil or paclitaxel when compared to the microtumor model (Fig 6E), demonstrating improvements in the ability of the microtumors to predict the physiological response. The morphology and size of the breast cancer microtumors reinforces the growth and invasion measurements (Fig 5B and 5C), and it reveals additional information regarding the other cell types in culture. Specifically, paclitaxel inhibited recruitment of the tubule networks to the breast cancer microtumors by stabilizing the microtubules and preventing centrosome reorientation, which is necessary for cell migration [53]. Vascular recruitment and invasion is indicative of angiogenesis, and its inhibition is another promising anti-tumor strategy. Thus, microtumors provide a mechanism for evaluating tumor cells directly, as well as other cell populations in the growing tumor tissue.

In summary, we have developed an *in vitro* microtumor of breast tumor cell spheroids, endothelial cells, and stromal stem cells in a basement membrane matrix under physiological tumor-aligned microenvironmental conditions for cell and matrix architecture, low pH, low glucose, and hypoxic conditions. Besides providing the correct tumor microenvironment, this model shows reduced growth similar to the *in vivo* situation over that observed with 2D and 3D monoculture. The findings with two known chemotherapeutic agents were physiologically similar to published xenograft data suggesting the ‘proof of principle’ of the model. Our combined use of three cell types and tumor microenvironment conditions provides the appropriate tissue and cell architecture to additively improve the model over those previously developed.

## Supporting Information

S1 Fig Breast cancer cell invasion is evaluated by measuring the increase in area for breast cancer cells in the microtumor using ImageJ. (TIF)

S2 Fig The stepwise process for the *in vitro* microtumor model for evaluating breast cancer progression. (TIF)

S3 Fig Breast cancer cells exhibit differential morphologies in 2D culture, 3D culture and microtumor models. (TIF)

S1 Striking Image Caption (DOCX)
