# Supplementary figures and images for: In Vitro Microtumors Provide a Physiologically Predictive Tool for Breast Cancer Therapeutic Screening
Source: PLoS One. 2015 Apr 9;10(4):e0123312. doi: 10.1371/journal.pone.0123312 (PMC4391795; doi:10.1371/journal.pone.0123312)

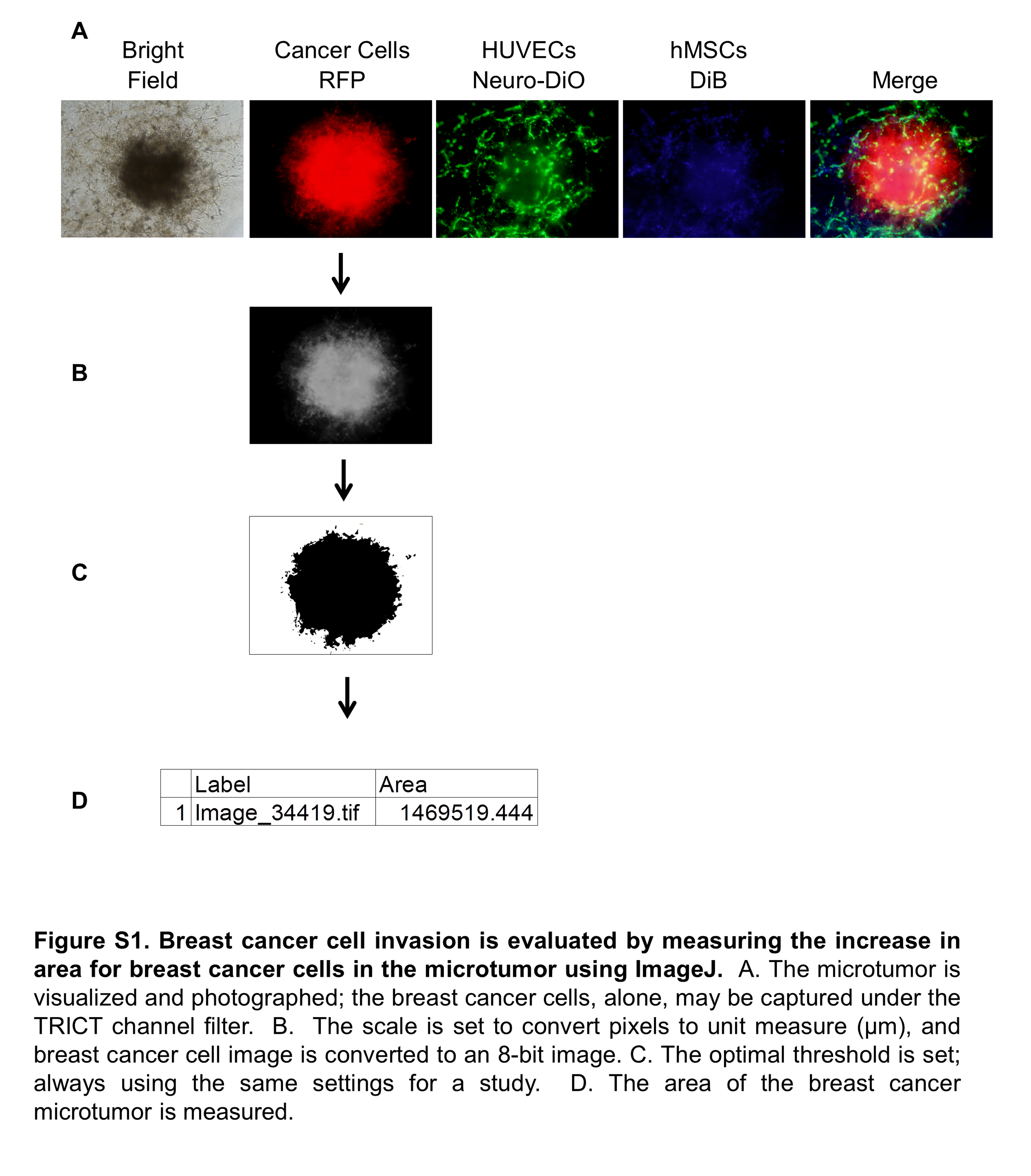

Supplement: S1 Fig — (TIF) [file pone.0123312.s001.tif]

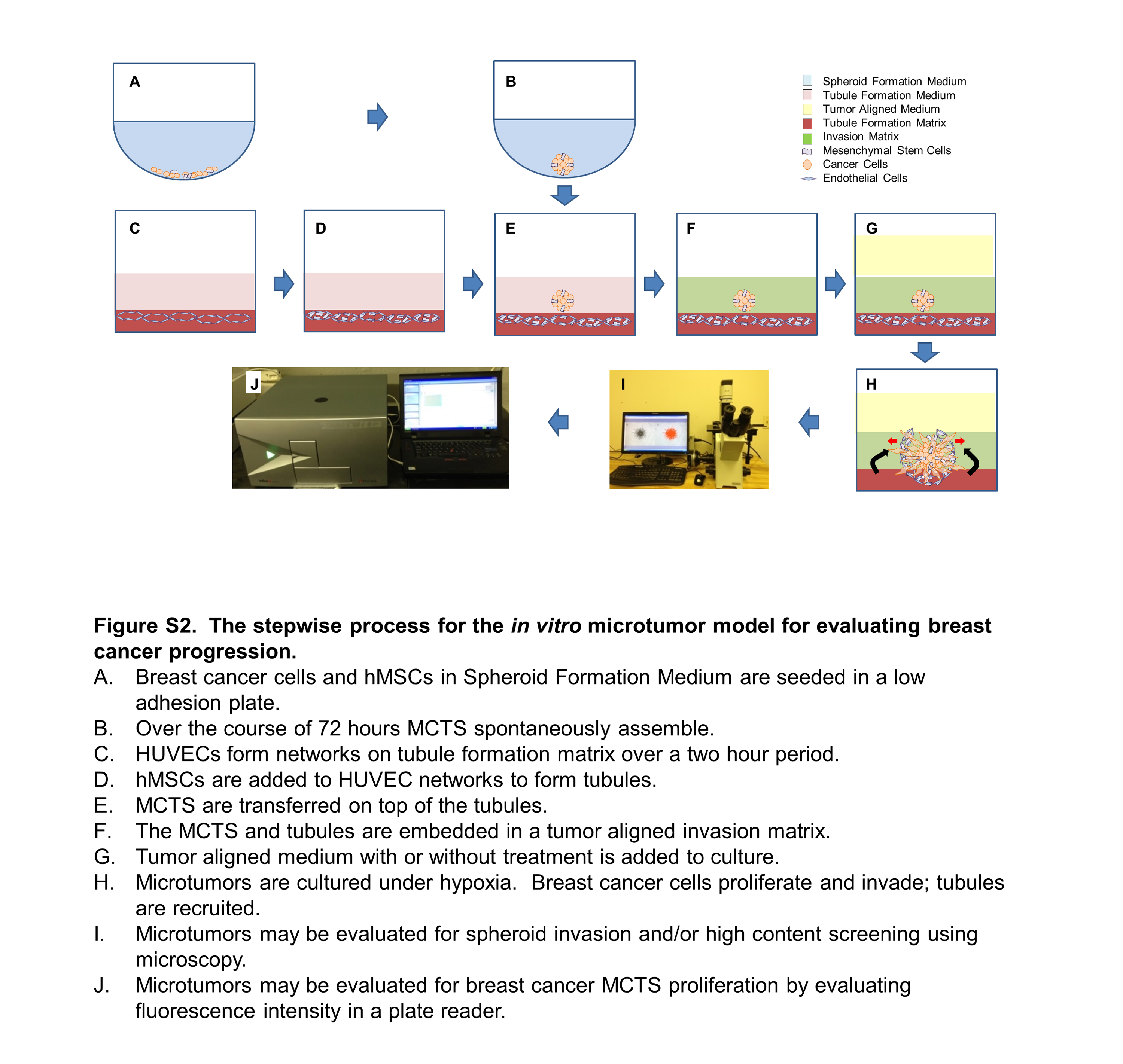

Supplement: S2 Fig — (TIF) [file pone.0123312.s002.tif]

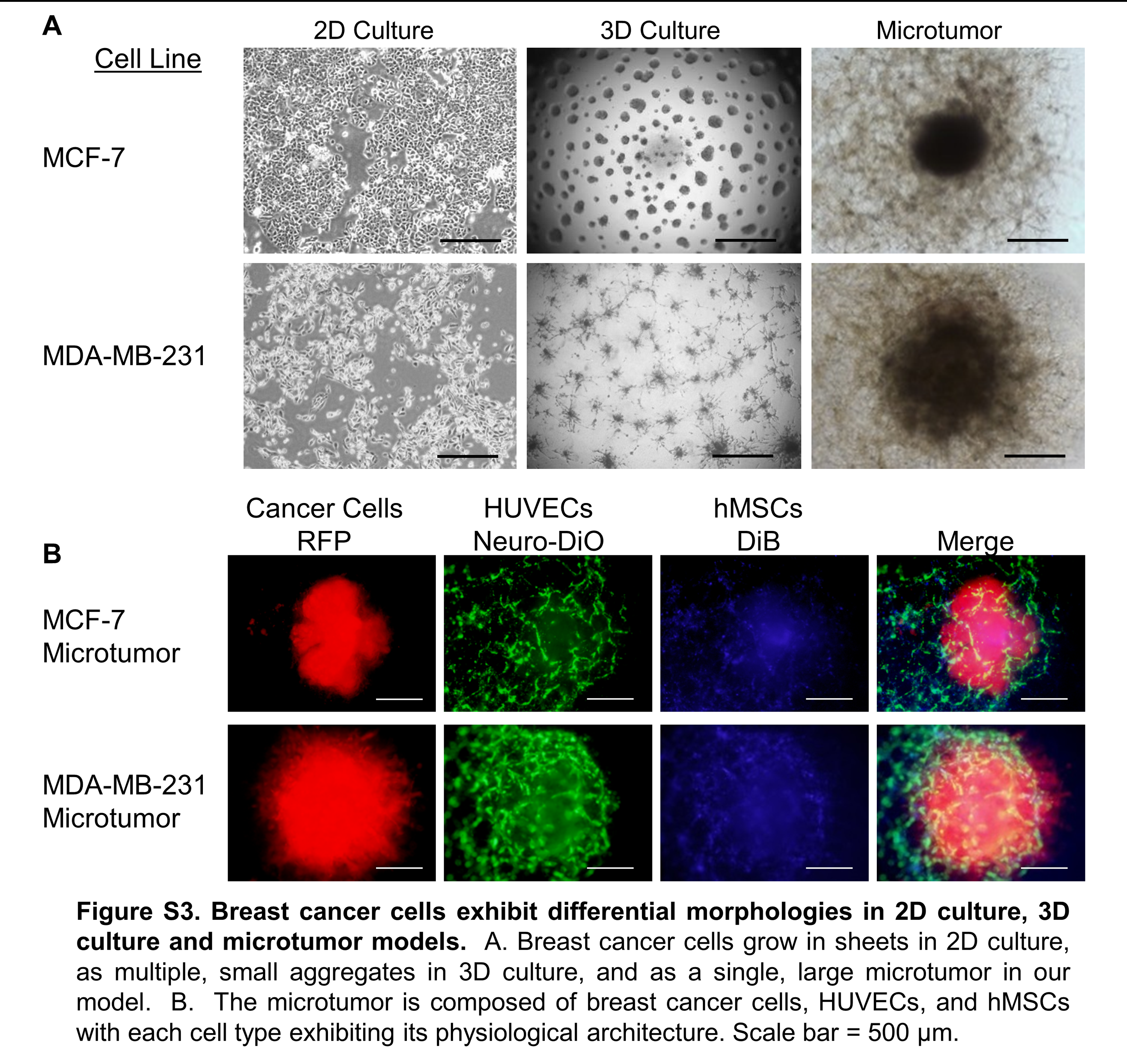

Supplement: S3 Fig — (TIF) [file pone.0123312.s003.tif]
