# Supplementary material for: In Vitro Microtumors Provide a Physiologically Predictive Tool for Breast Cancer Therapeutic Screening
Source: PLoS One. 2015 Apr 9;10(4):e0123312. doi: 10.1371/journal.pone.0123312 (PMC4391795; doi:10.1371/journal.pone.0123312)
Supplement: S1 Striking Image Caption — (DOCX) [file pone.0123312.s004.docx]

Supporting Information:

Striking Image caption:

*In vitro* microtumors recreate *in vivo* tumor architecture.

Description:

Human breast cancer microtumors are assembled in a tumor-aligned extracellular environment using MCF-7 human breast cancer cells (red), human umbilical vein endothelial cells (green), and human mesenchymal stem cells (blue).
